# Surgical strategies protecting against right ventricular dilatation following tetralogy of Fallot repair

**DOI:** 10.1186/s13019-018-0702-0

**Published:** 2018-01-22

**Authors:** Amr A. Arafat, Elatafy E. Elatafy, Sahar Elshedoudy, Mahmoud Zalat, Neamet Abdallah, Ahmed Elmahrouk

**Affiliations:** 1 Cardiothoracic Surgery Department, Tanta University, Al-Geish Street, Tanta, 31527 Gharbya Egypt; 2 Cardiology Department, Tanta University, Tanta, Egypt; 3 Cardiothoracic Surgery Department, Misr Children Hospital, Cairo, Egypt; 4 Cardiology Department, Misr Children Hospital, Cairo, Egypt

**Keywords:** Tetralogy of Fallot repair, Pulmonary leaflets sparing, Infundibular preservation, Tricuspid valve repair

## Abstract

**Background:**

Right ventricular (RV) volume overload increases morbidity and mortality after tetralogy of Fallot (TOF) repair. Surgical strategies like pulmonary leaflets sparing and tricuspid valve repair at time of primary repair may decrease RV overload. Our objective is to evaluate early and midterm results of pulmonary leaflets sparing with infundibular preservation and tricuspid valve repair in selected TOF patients with moderate pulmonary annular hypoplasia.

**Methods:**

From 2011 to 2016; 46 patients with TOF and moderate pulmonary annular hypoplasia had surgical repair with sparing of the pulmonary valve leaflets. Concomitant tricuspid valve repair was performed in 33 patients (71.8%). Mean age was 13.1 ± 4.8 months, 68% were males (*n* = 31) and mean weight was 9.5 ± 2.3 kg. Preoperative McGoon ratio was 1.9 ± 0.4 and pulmonary valve z-score ranges from − 2 to − 3. Preoperative pressure gradient of RVOT was 80.9 ± 7.7 mmHg and 10.9% had minor coronary anomalies (*n* = 5).

**Results:**

All repairs were performed through trans-atrial trans-pulmonary approach. 87% had pulmonary valve commissurotomy (*n* = 40). Mean cardiopulmonary bypass time was 71 ± 6.3 min and ischemic time 42.4 ± 4.9 min. Hospital mortality occurred in 4.3% (*n* = 2). Mean RVOT pressure gradient decreased significantly postoperatively (28.8 ± 7.2 mmHg, *p*-value< .001) and at the last follow up (23.6 ± 1.8 mmHg, *p*-value< .001). Pulmonary regurgitation progressed by one grade in 2 patients compared to the postoperative grade. 1 patient (2.5%) had late mortality and reintervention was required in 5 patients (12.5%).

**Conclusion:**

Pulmonary leaflets sparing, and tricuspid valve repair are safe for TOF repair with no added morbidity or mortality. These procedures could contribute to reducing right ventricular volume overload over time after TOF repair.

## Background

Severe pulmonary valve regurgitation following tetralogy of Fallot (TOF) repair leads to right ventricular (RV) volume overload. Volume overload of the right ventricle is aggravated with any associated tricuspid regurgitation and it is considered the major cause of function impairment and increased morbidity and mortality in those patients [1–3]. Several techniques have been proposed to alleviate the acute and chronic right ventricular volume overload after TOF repair and recently pulmonary valve sparing procedure has gained popularity [4–7]. Pulmonary valve sparing could decrease right ventricular volume overload with the benefit of preserving the growth potential of the pulmonary valve. However, it bears the risk of residual pulmonary valve stenosis with increased right ventricular pressure afterload especially when performed in patients with moderate and severe annular hypoplasia. Recently repair of more than moderate degree of tricuspid valve regurgitation at time of pulmonary valve replacement was found to be associated with improved patients’ functional status [8]. The objective of this study is to evaluate early and midterm effects of pulmonary leaflets sparing and tricuspid valve repair in TOF repair in selected patients with moderate pulmonary annular hypoplasia on right ventricular outflow tract (RVOT) pressure gradient, degree of pulmonary regurgitation, tricuspid valve regurgitation and reoperation.

## Methods

### Patients population

This retrospective cohort study included 46 patients with tetralogy of Fallot who were operated by the same surgical team from January 2011 to March 2016. Selection of patients was based on the preoperative pulmonary annulus z-score, the study included all consecutive TOF patients who had moderate pulmonary annular hypoplasia with z score ranges from − 2 to − 3 and amenable for pulmonary leaflets preservation. Patients with severe pulmonary annular hypoplasia, pulmonary atresia, absent pulmonary valve or discontinuation of the pulmonary arteries were excluded from the study. Moreover, patients with moderate annular hypoplasia (z-score from − 2 to − 3) with dysplastic pulmonary valve had mono-cusp pulmonary valve reconstruction and were excluded from the study. The study was approved by the ethical committee and patients’ consent was waived due to the retrospective nature of the study.

### Preoperative data collection

Patients’ charts and preoperative echocardiography were retrospectively reviewed to collect the preoperative patients’ characteristics, pulmonary annulus z-score, RVOT pressure gradient, associated lesions, prior shunts and the preoperative rhythm. McGoon ratio were measured by echocardiography as the sum of the diameters of immediately pre-branching left and right pulmonary arteries to descending aorta just above level of diaphragm.

### Surgical technique

Surgical repair of TOF was performed through full median sternotomy whether primary or redo sternotomy. After aorto-bicaval cannulation, cardiopulmonary bypass was commenced with cardioplegic arrest and cold blood cardioplegia was repeated every 25 min. All operations were performed through a trans-atrial trans-pulmonary approach. Right atriotomy was performed and venting was done through the inter-atrial septum. Splitting of the obstructing bundles in the infundibulum with very minimal resection was performed to preserve the right ventricular outflow tract geometry. Closure of the ventricular septal defect was done with Gore-Tex patch using polyprolene 6/0 continuous sutures. Pulmonary arteriotomy was performed to complete the resection of the obstructing bundles. Pulmonary valvotomy was done in most patients and in case of borderline annulus not passing 2 sizes less than the expected Hegar dilator for the body surface area, the incision was extended few mms though the annulus keeping the valve in situ. Pulmonary valvotomy was performed at the site of the fused leaflets. Commissural suspension was performed in 2 patients. Closure of the incision was performed using glutaraldehyde treated pericardial patch. In patients with more than mild degree of tricuspid regurgitation identified by the preoperative echocardiography and confirmed by intraoperative saline test, tricuspid repair was performed. Tricuspid repair was done by closing the commissure between anterior and septal leaflets in case of sufficient septal leaflet tissues or by bicuspidization technique in case of rudimentary septal leaflet. RV and PA pressures were directly measured after weaning from cardiopulmonary bypass and pressure gradient of 30–35 mmHg was accepted.

### Postoperative clinical data

Postoperative clinical data including duration of intensive care unit (ICU) and hospital stay were collected. Patients were followed clinically and echocardiography after discharge and mean duration of follow up was collected.

### Echocardiographic follow up

Transesophageal echocardiography (TEE) was performed after TOF repair in all patients to evaluate the adequacy of the repair. All patients had transthoracic echocardiography pre-discharge. Further follow up was performed by the cardiologists and the last echocardiographic data were collected including the degree of pulmonary and tricuspid valve regurgitation and RVOT pressure gradient. Time to reintervention, causes and types “whether surgical or catheter based intervention” were reported.

### Study outcomes

Pulmonary regurgitation (PR) was recorded and graded from 0 to 4 (0, none; 1, trivial; 2, mild; 3, moderate and 4, severe). RVOT pressure was reported from last echocardiographic evaluation and compared to the preoperative and pre-discharge values. Recurrence of tricuspid regurgitation was reported at follow up.

### Statistical analysis

Continuous variables were presented as mean ± standard deviation, median and range and categorical variables as numbers and percent. Continuous variables were compared using paired *t*-test for normally distributed variables and categorical variables with Fisher’s exact test. Changes in RVOT pressure were plotted against time and random effect model was used to test the significance of the change. Logistic regression was used to identify predictors of the use of trans-annular patch and odds ratio were reported. All analyses were performed using STATA 14 statistical software (Statacorp, Texas, USA). *P*-value less than 0.05 was considered significant.

## Results

### Patients’ characteristics and operative data

Forty- Six patients had TOF repair with pulmonary leaflets sparing and infundibular preservation. All patients had moderate pulmonary annular hypoplasia with z-score ranged from − 2 to − 3. Mean age at time of repair was 13.1 ± 4.8 months (median age = 11 months). Trans-annular patch (TAP) was required in 23.9% (*n* = 11) and pulmonary valve commissurotomy in 86.96% (*n* = 40) patients. Five patients had minor coronary anomaly defined as small conal branch crossing the RVOT and 1 patients had the left anterior descending artery originating from the right coronary artery. Most common pulmonary valve morphology was bicuspid valve in 56.5% (*n* = 26) and tricuspid valve in 37% (*n* = 17). Tricuspid valve repair was performed concomitantly in 72% patients (*n* = 33) to reduce postoperative right ventricular volume overload (Table 1).

**Table 1:** Preoperative and operative data

Patients’ characteristic	Value
Age (months)	
Mean ± SD	13.1 ± 4.8
Median	11
Range	8–26
Male	31 (67.4%)
Weight (Kg)	
Mean ± SD	9.5 ± 2.3
Median	9
Range	6–16
Associated lesions:	
ASD	4 (8.7%)
PDA	7 (15.2%)
PLSVC	1 (2.2%)
McGoon Ratio	
Mean ± SD	1.9 ± 0.14
Median	1.9
Range	1.7–2.2
RVOT pressure Gradient (mmHg)	
Mean ± SD	80.9 ± 7.7
Median	80
Range	65–97
Prior MBT shunt	8 (17.4%)
Coronary anomalies	
No	40 (86.96%)
Minor	5 (10.87%)
Major	1 (2.17%)
Preoperative Rhythm	
Sinus	44 (95.66%)
Partial HB	1 (2.17%)
Complete HB	1 (2.17%)
TAP	11 (23.9%)
Cardiopulmonary bypass time (min)	
Mean ± SD	71.2 ± 6.3
Median	70
Range	55–94
Ischemic time (min)	
Mean ± SD	42.4 ± 4.9
Median	42
Range	35–62
Pulmonary valve morphology	
Unicuspid	1 (2.17%)
Bicuspid	26 (56.52%)
Tricuspid	17 (36.96%)
Indeterminate	2 (4.35%)
Pulmonary valve commissurotomy	40 (86.96%)
TV repair	33 (71.74%)

(Continuous variables are expressed as mean ± SD, median and range) Categorical variables are presented as number and (percent)) *ASD* Atrial septal defect, *PDA* Patent ductus arteriosus, *PLSVC* Persistent left superior vena cava, *RVOT* Right ventricular outflow tract, *MBT* Modified Blalock Taussig shunt, *HB* Heart block, *TAP* Trans-annular patch

### Clinical outcomes

Mean ICU stay was 3.1 ± 1.2 days. Hospital mortality occurred in 4.4% (*n* = 2) patients, 4 patients lost for follow up and late mortality occurred in 1 patient. Reintervention either surgical or catheter based was performed in 5 patients and indications of reintervention were: RVOT obstruction, restrictive ventricular septal defect (VSD), complete heart block, left pulmonary artery stenosis and diaphragmatic paralysis. The interventions performed were resection of the right ventricular obstructing bundle, open VSD closure, trans-venous pacing, trans-catheter stenting of the left pulmonary artery and diaphragmatic plication respectively. Mean time from primary repair to reintervention was 0.94 ± 0.6 years (median = 1.2 years) (Table 2).

**Table 2:** Early and late clinical outcomes

Clinical outcomes	Value
ICU stay (days)	
Mean ± SD	3.1 ± 1.2
Median	3
Range	2–8
Hospital stay (days)	
Mean ± SD	8.9 ± 2.4
Median	8
Range	4–18
Hospital mortality	2 (4.35%)
Late Mortality	1/40 (2.5%)
Late reoperation	5/40 (12.5%)
Tricuspid Regurgitation (≥grade II)	1 (2.5%)
Rhythm at follow up	
Sinus	38 (95%)
Nodal	1 (2.5%)
Complete Heart block	1 (2.5%)

(Continuous variables are expressed as mean ± SD, median and range) Categorical variables are presented as number and (percent)

### Pulmonary valve and RVOT

**Fig. 1:**
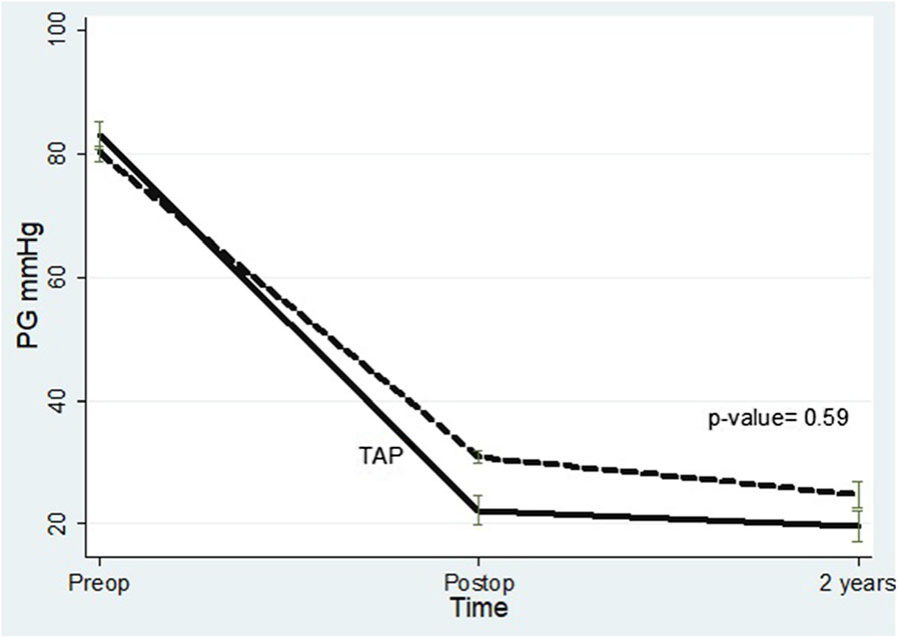
Change in RVOT pressure in patients with and without transannular patch. Values are presented as preoperative, postoperative and at 2 years follow up

Mean time to the latest echocardiographic follow up was 3.85 ± .85 years. RVOT pressure gradient dropped significantly postoperatively compared to the preoperative gradient (*p*-value < 0.0001). The decline continued during follow up (Table 3). The decrease in RVOT pressure between patients with and without TAP was comparable (*p* = .59) (Fig. 1).

Pulmonary regurgitation regressed by one grade in 10 patients compared to the pre-discharge echocardiography and progressed by one grade in 2 patients, both had TAP. (*p*-value < 0.001) (Table 4).

Low body weight at time of repair predicted of the use of the TAP (*p* = 0.03) (Table 5) and the use of TAP was significantly associated with postoperative PR (*p* = .003).

**Table 3:** Comparison of the predischarge and last follow up RVOT pressure gradient in (mmHg) to the preoperative measurement

Timing	Mean ± SD	Standard Error	95% confidence interval	*p*- value
Pre-operative	80.9 ± 7.7	1.14	78.6–83.2	< 0.0001
Predischarge	28.8 ± 7.2	1.07	26.6–30.9	
Last follow up	23.6 ± 11.5	1.8	19.9–27.2	0.01

**Table 4:** Change in the pulmonary regurgitation (PR) grade at the last follow up compared to the predischarge grade (Numbers in the table present patients number)

Degree of predischarge PR	Degree of PR at the last follow up				
	0	1	2	3	total
0	4	1	0	0	5
1	5	18	0	0	23
2	0	5	5	1	11
3	0	0	0	1	1
Total	9	24	5	2	40

**Table 5:** Predictors of the use of transannular patch (TAP) in patients with moderate pulmonary annular hypoplasia

	Odds ratio	Std.Err	Z statistics	*P*-value	95% Confidence interval
Age	1.24	0.63	0.42	0.67	0.46–3.38
Weight	0.15	0.14	−2.06	0.03	0.26–0.9
RVOT pressure gradient	1.14	0.09	1.6	0.1	0.97–1.32

### Tricuspid valve

Tricuspid valve repair was performed in 71.8% of the patients for moderate and severe degree of tricuspid regurgitation at time of primary TOF repair (*n* = 33). All patients had no degree of tricuspid regurgitation postoperatively. During follow up, 8 patients (20%) developed mild degree of tricuspid regurgitation and 1 patient (2.5%) had moderate tricuspid regurgitation. Tricuspid regurgitation significantly occurred in patients with no prior tricuspid valve repair (*p*-value = 0.002).

## Discussion

Since the first repair of tetralogy of Fallot which was performed through a large right ventricular incision with the use of trans-annular patch, [9] several surgical strategies have been developed to reduce the drawbacks of TOF repair especially right ventricular volume overload. The deleterious effect of chronic pulmonary regurgitation following TOF repair has shifted the paradigm of TOF management from strategy aiming mainly to relieve pulmonary stenosis to one aiming at preserving the pulmonary valve. The monocusp valve reconstruction with autologous pericardium or polytetrafluoroethylene was attempted to decrease the degree of pulmonary regurgitation postoperatively [10]. Despite the reported success of the technique by some authors, [11, 12] it doesn’t gain a wide acceptance because of lack of growth potential of the prosthetic material and the high reoperation rate. Biological material was used to reconstruct the monocusp pulmonary valve to preserve the growth potential however, progressive pulmonary regurgitation was noted during follow up [5]. Our conservative technique is based on preserving the native pulmonary valve leaflets in patients presenting with moderate annular hypoplasia (z-score − 2 to − 3) by performing pulmonary valvotomy, limited arteriotomy and keeping the valve in place to preserve its natural geometry. This strategy was not associated with residual pulmonary valve stenosis and RVOT pressure gradient was effectively reduced both postoperatively and at follow up. Pulmonary regurgitation has been reduced by one grade over the follow up compared to the pre-discharge grade and no reoperation on pulmonary valve was required in a mean follow up of 3.85 ± .85 years although long term follow up is recommended. The changes of pulmonary regurgitation in our series could be explained by the growth potential of preserved pulmonary valve leaflets. Pulmonary valve z-score 4 or less was found to be a predictor of recurrent RVOT obstruction in other series [13] and our current strategy is to preserve the pulmonary valve in patients without severe annular hypoplasia. Hoashi et al., [14] found that pulmonary valve z-score was not a predictor of recurrent ROVT obstruction but z-score less than − 2 was a predictor of progressive pulmonary regurgitation which was not found in our study, the degree of pulmonary regurgitation regressed by one grade in 10 patients during the follow up. The most common pulmonary valve morphology encountered was a bicuspid valve followed by tricuspid morphology. This finding is in concordance with Vida et al., [7] who found that bicuspid and tricuspid pulmonary valve morphology were amenable for preservation on contrary to the unicuspid morphology. We encountered 1 patient with unicuspid valve and 2 patients with indeterminate morphology due to fusion of the commissures. Those patients had non-progressing grade I PR postoperatively and at the last echocardiographic follow up but no definite conclusion about the suitability of those valves for repair can be drawn due to the small patients’ number. Hoashi et al., [14] had 66.7% bicuspid pulmonary valve in their series of 84 patients and bicuspid pulmonary valve was a significant predictor for increasing pulmonary regurgitation during the long term follow up.

Despite pulmonary regurgitation is a major cause of deterioration of the right ventricular function, several other factors can also contribute to functional deterioration following TOF repair [1, 2, 15]. Trans-ventricular TOF repair could contribute to the progressive right ventricular dilatation. Lee et al.; found no difference in outcome between patients who had traditional versus limited right ventriculotomy [16]. In our series, all repairs were performed through trans-atrial trans-pulmonary approach with minimal resection but rather splitting of the obstructing infundibular bundles. This strategy yielded good short-term results with 1 patient had complete heart block and required a permanent pacemaker insertion and 1 patient had late operation due to persistent RVOT obstruction. No ventricular arrhythmia was reported in any of our patients.

Postoperative tricuspid regurgitation was found in other series to be a predictor of late morbidity [17]. We follow a rigorous strategy to prevent postoperative tricuspid valve regurgitation by routinely performing repair of valve with more than mild degree of regurgitation post TOF repair. 72% of our patients required tricuspid valve repair at time of primary TOF repair. This high percentage of patients requiring concomitant tricuspid repair in our series could be attributed to the relatively older age of our patient population compared to the other published series. In older children, the right ventricle is subjected to long period of overload, with a resultant decrease in the compliance of the RV and annular dilatation. We also included repair of the regurgitation resulted from septal leaflet distortion during closure of the VSD. Tricuspid repair significantly halted the progress of tricuspid regurgitation at follow up. However long-term follow up is required to identify the effect of this procedure on right ventricular function.

Limited trans-annular pulmonary patch was required in 11 patients. We have observed no difference postoperatively and at follow up between patients with and without TAP regarding the decrease of the RVOT pressure gradient. Follow up was complete in 9 patients with TAP, 2 had progression of the degree of PR by one grade and the degree of PR remained stationary in 6 patients. On the other hand, the degree of PR improved in patients without TAP (*n* = 9) by one grade. These results are consistent with Sen et al., [5] who found improvement in PR with valve preservation or reconstruction by biological material compared to TAP. Similar to other reports, TAP was significantly associated with postoperative PR and low weight predicts the use of the patch preoperatively [18, 19]. In contrast to our findings, Simon et al. found that limited trans-annular patch yielded similar results compared to annular sparing as regard to the degree of pulmonary regurgitation and right ventricular enlargement [20]. In summary, pulmonary leaflet sparing during TOF repair is associated with significant reduction of RVOT pressure without causing significant pulmonary regurgitation. Tricuspid valve repair halted the progression of tricuspid valve regurgitation. The use of the trans-annular patch is associated with increased pulmonary regurgitation compared to patients without TAP.

### Study limitations

The study is retrospective in nature which bears all the drawbacks of retrospective studies. Echocardiography was used in follow up which is not the standard diagnostic tool for evaluation of the pulmonary valve and right ventricular function and MRI is a better tool for assessment of the right ventricular function [21]. Another drawback is the short duration of follow up and long term follow up is recommended to determine the durability of the pulmonary and tricuspid valve repair and their effect on the right ventricular function.

## Conclusion

Pulmonary leaflets sparing and tricuspid repair can be safely performed at time of primary TOF repair. Pulmonary leaflets sparing doesn’t result in significant pulmonary regurgitation post TOF repair and tricuspid repair protects against future development of tricuspid regurgitation. The procedure has favorable short and midterm results and low reoperation rate. Trans-annular patch is associated with increased postoperative pulmonary regurgitation and its progression and it should be avoided when possible.

## Abbreviation

ASD: Atrial septal defect
HB: Heart block
ICU: Intensive care unit
MBT: Modified Blalock Taussig shunt
PDA: Patent ductus arteriosus
PLSVC: Persistent left superior vena cava
PR: Pulmonary regurgitation
RV: Right Ventricle
RVOT: Right ventricular outflow tract
TAP: Trans-annular patch
TEE: Transesophageal Echocardiography
TOF: Tetralogy of Fallot
TR: Tricuspid regurgitation
VSD: Ventricular septal defect
